# Snake in the heart: a rare inferior vena cava to pulmonary artery thrombus in pregnancy

**DOI:** 10.1093/ehjcr/ytag119

**Published:** 2026-02-24

**Authors:** Qayoom Yousuf, Aamir Rashid, Sameer Purra, Naseer Choh

**Affiliations:** Department of Cardiology, Sher-i-Kashmir Institute of Medical Sciences, Srinagar, Jammu and Kashmir 190011, India; Department of Cardiology, Sher-i-Kashmir Institute of Medical Sciences, Srinagar, Jammu and Kashmir 190011, India; Department of Cardiology, Sher-i-Kashmir Institute of Medical Sciences, Srinagar, Jammu and Kashmir 190011, India; Department of Radiology, Sher-i-Kashmir Institute of Medical Sciences, Srinagar, Jammu and Kashmir 190011, India

## Case description

**Figure 1:**
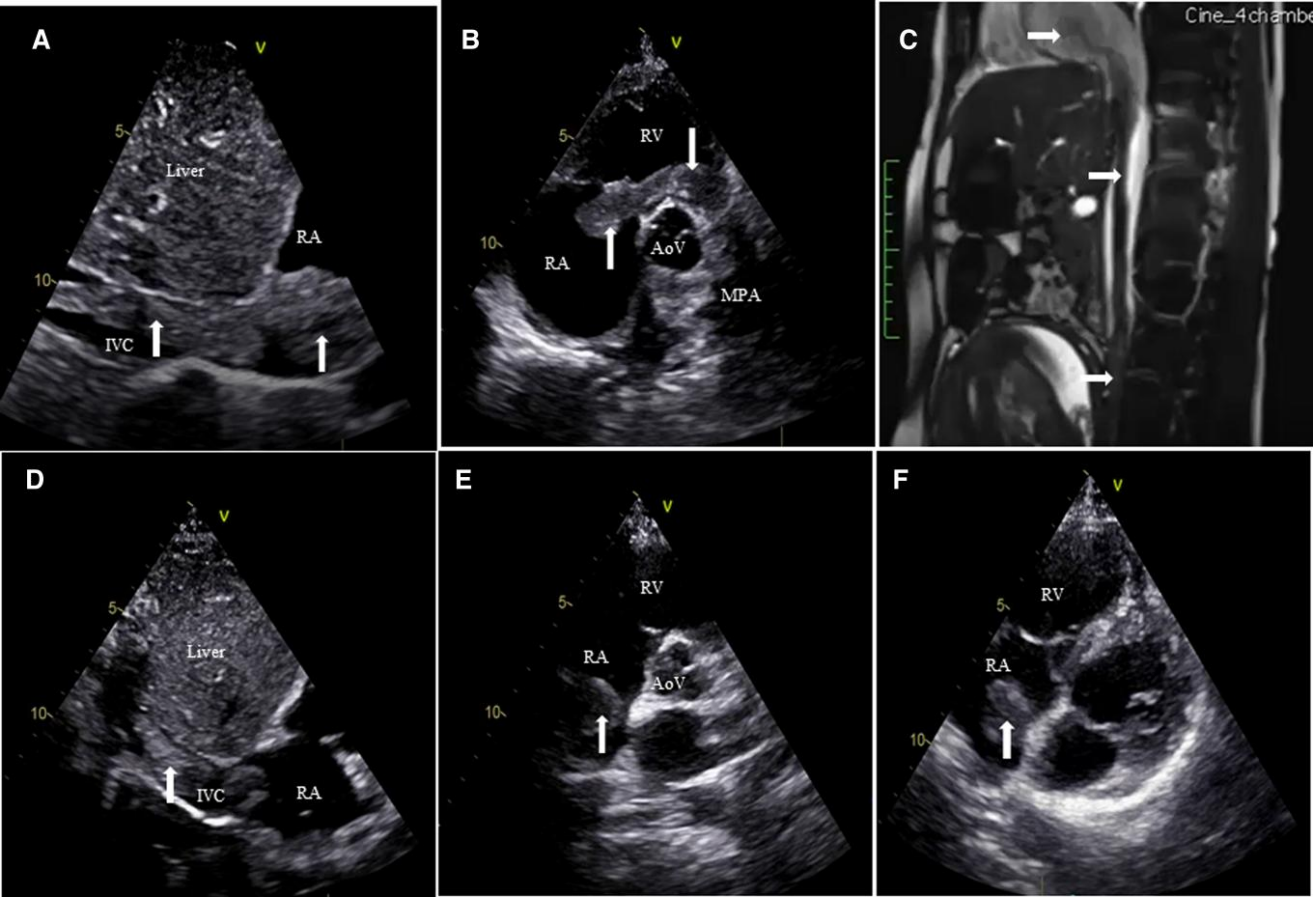
White arrows represent the clot. (*A*) Subcostal echo view showing serpiginous thrombus entering right atrium via inferior vena cava. (*B*) Parasternal short axis echo view—thrombus prolapsing across tricuspid valve into right ventricle and then across the pulmonary valve into main pulmonary artery. (*C*) Magnetic resonance imaging showing continuous filling defect from inferior vena cava to right atrium/right ventricle. (*D*) Subcostal inferior vena cava view showing decrease in the thrombus burden in inferior vena cava and right atrium after thrombolysis. (*E*) Parasternal short axis echo view showing resolution of clot in right ventricle and main pulmonary artery post thrombolysis. (*F*) Apical four-chamber view showing residual clot in right atrium.

A 24-year-old primigravida at 26 weeks gestation presented with progressive dyspnoea (NYHA II) and pedal oedema for 1 month. She had no prior significant medical history. Vital signs and oxygen saturation were stable. Laboratory tests showed normal haemoglobin, leukocyte and platelet counts, normal renal and hepatic functions, elevated D-dimer (1268 ng/mL), and mildly raised BNP (146 pg/mL). Echocardiography revealed a long serpiginous thrombus (white arrows) extending from the inferior vena cava (IVC) into the right atrium (RA), prolapsing across the tricuspid valve into the right ventricle (RV) and reaching the main pulmonary artery (MPA) (*Figure 1A* and *B*). Mild RA and RV dilatation was present without significant right ventricular systolic dysfunction, and the estimated systolic pulmonary arterial pressure was approximately 48 mmHg. Magnetic resonance imaging confirmed a continuous intraluminal filling defect tracking from the iliac system through the IVC into the right heart (*Figure 1C*).

Given the gestational age, extensive thrombus burden, and the substantial maternal–foetal risks associated with surgical embolectomy, the multidisciplinary team elected to administer systemic thrombolysis. Systemic thrombolysis was initiated with streptokinase administered via the femoral venous route, using a loading dose of 250 000 IU infused intravenously over 30 min, followed by a continuous infusion of 100 000 IU/h. Serial echocardiography demonstrated marked thrombus regression, with complete disappearance of the RV and MPA components and reduction of IVC and RA segments (*Figure 1D–F*). Owing to the extensive intracardiac thrombus burden and on-going thrombus dissolution on serial echocardiography, the infusion was continued for a total duration of 48 h. Following systemic thrombolysis, the patient was bridged by enoxaparin to oral anticoagulation with warfarin, which was continued during pregnancy under close maternal–foetal monitoring. Thrombophilia testing after 2 weeks showed reduced protein C levels while evaluation for protein S, lupus anticoagulant, anti-beta-2 glycoprotein antibodies, anticardiolipin antibodies, anti-nuclear antibody (ANA), Factor V Leiden mutation, and prothrombin gene mutation were negative. Warfarin was electively discontinued at around 36 weeks’ gestation and replaced with therapeutic enoxaparin, which was subsequently transitioned to intravenous unfractionated heparin in the peripartum period to permit rapid reversibility. She remained stable and delivered a healthy neonate by uneventful lower-segment caesarean section at 36 weeks. Postpartum anticoagulation was transitioned to rivaroxaban. In light of emerging evidence indicating minimal drug excretion into breast milk, the patient continued breastfeeding under close neonatal monitoring, with no observed adverse effects. Repeat thrombophilia testing at 12 weeks after delivery confirmed persistent protein C deficiency, and long-term anticoagulation was continued.

Pregnancy increases the risk of venous thromboembolism nearly six-fold, with peak risk postpartum.^1^ Underlying thrombophilias such as protein C deficiency may contribute to extensive clot burden, as in this case. Management is challenging because no definitive guidelines exist regarding the use of anticoagulation alone, thrombolysis or surgical embolectomy in pregnancy.^2^ However, several contemporary case reports, observational studies, and meta-analysis support a favourable role for thrombolysis in selected high-risk cases.^3^

This case highlights a rare continuous iliac–IVC–right-heart thrombus in pregnancy and demonstrates favourable outcomes with individualized thrombolysis-assisted reperfusion and anticoagulation-based management.

## Data Availability

Data will be made available on request.

## Notes

**Consent:** The authors confirm that written consent for submission and publication of this case report including images and associated text has been obtained from the patient in line with COPE guidance.
